# Psychosocial effects of social media on the Saudi society during the Coronavirus Disease 2019 pandemic: A cross-sectional study

**DOI:** 10.1371/journal.pone.0248811

**Published:** 2021-03-18

**Authors:** Mohammad Ahmed Hammad, Turki Mahdi Alqarni

**Affiliations:** Special Education Department, Faculty of Education, Najran University, Najran, Kingdom of Saudi Arabia; University of São Paulo, BRAZIL

## Abstract

The Coronavirus Disease 2019 (COVID-19) pandemic has been posing a substantial challenge to human survival and well-being, which rely on the actions and behaviors of individuals. It is essential that accurate information is distributed; however, misinformation has been spread via social media. Consequently, the resulting panic has to be addressed while putting essential public health measures in place. It is also important to explore the link between the social media exposure and well-being. Therefore, in the current study, we aimed to identify the levels of anxiety, depression, and social isolation among individuals during the COVID-19 pandemic. Additionally, we explored the relationship between exposure to misleading social media news and anxiety, depression, and social isolation. A cross sectional design was employed to collect data from 371 Saudi participants (aged 16–60 years), using the Generalized Anxiety Disorder-7, Centre for Epidemiological Studies Depression Scale, and de Jong Gierveld Loneliness Scale. Results showed that the prevalence of anxiety, depression, and social isolation was 47.82%, 47.57%, and 46.42%, respectively. Further, more than 83% of the participants reported using social media frequently during the pandemic. We found that exposure to misinformation via social media has a significant positive relationship with anxiety, depression, and social isolation. However, Due to the cross-sectional nature of this study it cannot be determined whether social media causes negative mental health outcomes, or if individuals experiencing greater depression, anxiety and social isolation turn to social media more than others, or if some third variable might explain both. Based on our findings, we present specific suggestions related to the COVID-19 pandemic to the government of Saudi Arabia. Minoring and filtering out misleading information with the cooperation of the World Health Organization (WHO) can promote the spread of accurate news in Saudi Arabia.

## Introduction

False information amplifies the challenges that humanity faces, such as the Coronavirus Disease 2019 (COVID-19) pandemic [1, 2]. Within weeks of the appearance of the virus in China, misleading rumors and conspiracy theories about its origin spread globally, coupled with stress and shock [3, 4]. This resulted in excessive storage and purchase of goods and facial masks [5]. The abundance of information, including false information, has been closely linked to the 21st century communication system, social media, which includes YouTube, Facebook, Twitter, Snap Chats, Instagram, WhatsApp—key applications that may thrive during the pandemic [6]. While staying at home during lockdown, people tend to interact extensively with friends and relatives through social media sites like (Facebook, Twitter, WhatsApp, etc …) to obtain information about the pandemic [7]. However, the information may be inaccurate and unreliable. Therefore, it is critical to monitor and reduce its adverse psychological and social effects on people, which can help them manage the pandemic better within communities [8, 9].

The dissemination of misleading and false information via social media has many negative psychological and social consequences for community members [10, 11]. In fact, Dr. Tedros Ghebreyesus, Director-General of the World Health Organization (WHO), said, “the wrong information about COVID-19 may be the most infectious disease,” and noted that misinformation about the COVID-19 pandemic has spread just as rapidly as the virus itself [12]. The stress and shock greatly affected the social and psychological well-being of the public [13, 14].

Social media has unprecedented dominance and power throughout the world, allowing the spread of misinformation and rumors that are often presented as facts by fearful citizens looking for a sense of security [15]. Times of crisis often increase anxiety and uncertainty about current events or information [2] and people attempt to resolve their doubts and anxieties through phone calls and social media to understand the situation better [16]. Further, several individuals (including the elderly) who have been forced to live alone during the lockdown became very dependent on social media to access information about the increasing number of infections and mortalities [17, 18]. This had several disadvantages (including increased stress, anxiety, tension, fear, and compulsive obsessions), particularly for those with mental disorders who rely on psychotherapy and psychiatric medication [19–23].

In the last few years, studies have been conducted, which indicated that social media exposure had little effect on mental health [24–26]; thus, the impact of social media on mental health is arguable [27]. However, other studies indicate that the spreading of misleading and fake information via social media led to many mental health problems, such as social isolation, poor individual relationships, family problems, post-traumatic stress disorder, anxiety, panic disorder, depression, and behavioral disorders, to social media news [6, 28–31]. Misleading social media information, in particular, has many psychological and social effects on individuals during crises and disasters, such as increased anxiety [32, 33], depression [34] and psychological stress [35]. The effect of the COVID-19 crisis on mental health [18, 36, 37] includes these negative psychological consequences [30, 38].

Therefore, public health measures should focus on limiting not only the spread of the COVID-19 pandemic but also the social media panic pandemic [39]. We need to respond quickly to the negative effects on individuals and society [39] and expose public rumors, perceptions, attitudes, and behaviors about COVID-19 discussed through social media [14].

The effective panic caused by social media can also be contained by disseminating the right information and supporting public health through social media. During the bans which were imposed due to the spread of COVID-19 in China, social media was used widely, and some governments monitored the information that was spread through such modes [14]. In this regard, social media provides an opportunity to clarify the reasons for imposing quarantine, and to reassure the public and supply practical advice to prevent rumors and panic [14]. Soltaninejad [40] reported that, in Iran, misleading information suggested that gargling with alcohol could prevent the spread of the pandemic. Consequently, many individuals abused alcohol, resulting in 2,200 related poisonings at the time. Accordingly, Llewellyn [41] pointed out that the best way to achieve a sense of psychosocial security during a crisis, including COVID-19, is to confront false information and rumors spread through social media by flooding the social media with accurate scientific information that is understood and shared easily, and by having certified specialists answer the questions of the public.

In Saudi Arabia, the society has witnessed a rise in social media sites, especially due to the increasing use of modern communication technologies. By January 2020, approximately 4.54 billion, 3.8 billion, and 5.19 billion people globally were using the internet, social networking sites, and mobile phones, respectively. Moreover, 2.78 billion individuals used their phones to surf social networking sites. Saudi Arabia ranks 17th in the rate of internet usage globally [42], and is at the forefront of active social media users internationally. In particular, 32.23 million individuals among its population of 34.218 million are Internet users. Of these, 25 million use social networks for an average of two hours and fifty minutes daily, mostly YouTube (76%), followed by WhatsApp (71%), Instagram (65%), Facebook (62%), and Twitter (58%) [43]. Social media has offered many benefits during closures and health isolation (or rather “social exclusion”). It continues to be the easiest method of accessing information and has played an important role in individuals’ ability to work from home and organize/join meetings, seminars, and conferences. However, we need to consider the risks it presents through misleading facts and rumors, especially in times of crises such as the current pandemic, as it may hold dire psychological, social, and health consequences for the society [10, 15, 40, 44, 45].

Considering the psychological, social, and behavioral effects of social media on the society during the COVID-19 pandemic, many countries, including Saudi Arabia, have implemented procedures such as taking legal action to counter the spread of rumors and misinformation. The Kingdom’s public prosecution has warned against spreading news from unknown sources and rumors through social media, especially those linked to the COVID-19 crisis; anyone who provides misleading information is subject to penal accountability [46].

The mere fact that social media is used widely in Saudi Arabia makes it necessary to identify the exact impact of misleading information on its citizens’ psychological and social well-being, in particular, on their levels of anxiety, depression, and social isolation. Similar research has not yet been undertaken in the Saudi society in relation to the pandemic. Therefore, we aimed to identify the level of generalized anxiety, depression, and social isolation among participants during COVID-19. Additionally, we examined the relationship of exposure to misleading information through social media with psychological and social well-being during the pandemic. We explored the following research questions:

Q1: *What is the level of generalized anxiety*, *depression*, *and social isolation among participants during COVID-19*?
Q2: *Is exposure to misleading news in social media related to the level of generalized anxiety*, *depression*, *and social isolation*?

## Materials and methods

### Participants

Three hundred seventy-one participants (male = 272, female = 99; age: 16–60 years) participated in this cross-sectional study in Najran, Saudi Arabia. Participants were invited to complete an online questionnaire one month after the spread of COVID-19 in Najran. The timeline of this study was concurrent with the lifting of restrictions applied nationally. Also, participants were randomly selected using the simple random sampling to provide equal and independent opportunity of selection for the sample.

### Procedures

This cross-sectional study was online conducted over six days (May 10–15 May 2020) after cases in Saudi Arabia reached almost two thousand and while curfew and social distancing measures were implemented by Government authorities. We obtained approval for our quantitative study from the Dean of Scientific Research at Najran University. The department of public ration’s and university media at Najran University has contacted the ministry of health in Saudi Arabia to distribute the survey link to all Saudi citizens. The ministry of health send messages with the survey link to Saudi citizens over six days. All participants completed the voluntary consent section in the questionnaire and were assured confidentiality. They completed the questionnaire on the website. The authors expected to obtain 450 responses within six days, but only 386 questionnaires were returned, of which 15 were excluded because they did not follow the questionnaire instructions. Consequently, the total number of questionnaires used for analysis was 371.

### Measurements

We administered the following three reliable scales with minor adaptions: the Generalized Anxiety Disorder-7 (GAD-7) [47], Centre for Epidemiological Studies Depression Scale (CES-D-10) [48], and De Jong Gierveld Loneliness Scale (DJGLS) [49].

#### Generalized anxiety disorder questionnaire

The GAD-7 is a seven-item questionnaire created to identify possible causes of generalized anxiety disorder and to measure the severity of the symptoms [47]. It assesses anxiety symptoms based on diagnostic criteria (Diagnostic Standards A, B, and C of the Diagnostic and Statistical Manual of Mental Disorders, Fourth Edition [DSM-IV]) [50]. Respondents are asked how often they have been upset in the past two weeks, experiencing any of the seven main generalized anxiety disorder symptoms. The answer options are “*Not at all*,” “*Several days*,” “*More than half the days*,” and “*Almost every day*” and are recorded as 0, 1, 2, and 3, respectively. Therefore, GAD-7 scores range from 0 to 21, with scores of 5, 10, and 15 representing mild, moderate, and severe anxiety symptoms, respectively. The scale has demonstrated excellent internal consistency (Cronbach *α* = .92) and reliability (intraclass correlation = .83) [47]. Several studies have confirmed its internal consistency, test-retest reliability, convergence, construction, standards, and validation [51–53]. For the present study, the scale was translated from English to Arabic and then back-translated. It was presented to specialists in English and Arabic linguistics to ensure the accuracy of the translation and linguistic formulation. Furthermore, the reliability of the data collected with the Saudi version of the GAD-7 was good (Cronbach α = .87), consistent with previous studies in which it ranged from .74 to .94 [51, 54].

#### Centre for epidemiological studies depression scale

The CES-D-10 is a 10-item Likert scale questionnaire that measures the occurrence of depressive symptoms over the week preceding the assessment [48]. It includes 10 elements, with three on depressive affect, five on physical symptoms, and two on positive affect. Among the 10 elements, eight assess the positive symptoms and two (Item 5 and 8) assess the negative symptoms of depression. The answer options for each item are presented on a four-point Likert scale ranging from “*rarely or none of the time*” (0) to “*all the time*” (3). Moreover, Item 5 and 8 are reverse scored as they are worded positively. Total scores range from 0 to 30, with higher scores indicating a greater severity of symptoms.

For the present study, the scale was translated (English to Arabic) and subsequently back-translated. We presented it to specialists in English and Arabic linguistics to ensure the accuracy of the translation and linguistic formulation. In the present study, the Saudi version showed adequate reliability and viability, with good internal consistency (Cronbach α = .81).

#### De Jong Gierveld loneliness scale

The six-item DJGLS aims to assess the social (example statement: “*there are many people I can completely trust*”) and emotional (example statement: “*I feel a general sense of emptiness*”) aspects of isolation between individuals, with three elements each. Response options are presented on a four-point Likert scale ranging from 1 (Strongly Disagree) to 5 (Strongly Agree). The original version’s Cronbach α was 0.76, which was consistent with many previous studies [55, 56]. In the present study, the scale was translated from English to Arabic, and back-translated. It was presented to specialists in English and Arabic linguistics to ensure the accuracy of the translation and linguistic formulation. The Saudi version of the DJGLS exhibited adequate reliability and viability in the present study, with good internal consistency (Cronbach *α* = .78).

#### Covariates

The following covariates were included in this study: gender, Marriage (Married and No married), age (16–25, 26–35, 36–50, 50–60 and More than 60), educational level (Less than secondary, Diploma / Secondary, Bachelor, Postgraduate), Living area(urban and rural), self-rated health (Excellent, Good, Sick).

### Data analysis

We applied descriptive and inferential statistics to analyze the data. The descriptive statistics included frequency, percentage, average and standard deviation; these were analyzed using SPSS 21 [57]. To address the first research question, we conducted a univariate analysis to compare the differences between participants’ characteristics based on social media exposure. Subsequently, we used multivariate analysis to examine the influence of social media exposure on the participants’ anxiety, depression, and social isolation. We used linear regression to examine whether the exposure to misleading news in social media is related to the level of generalized anxiety, depression, and social isolation.

## Results

Table 1 shows the frequency and percentage of participants’ social media exposure during the COVID-19 pandemic. Participants were predominantly male (73%) and approximately 60% were married. The groups with the highest representation were those aged 36–50 years (45.3%), those pursuing postgraduate education (48%) and those living in urban areas (80%). Contrarily, people aged over 60 years, those without secondary education, and those living in rural areas were the least represented groups in this study. Furthermore, most of the participants were in excellent health.

**Table 1:** Participants’ characteristics and social media exposure.

Variables	N	Social media exposure			
		Frequently	Sometimes	Less	P-value
**Overall**	371 (100)	238 (64.27%)	105 (28.39%)	28 (7.34%)	
**Gender**					
Male	272 (73.3%)	199 (73.16%)	64 (23.52%)	9 (3.30%)	<0.001
Female	99 (26.7%)	77 (77.77%)	17 (17.17%	5 (5.05%)	
**Marriage**					
Married	219 (59.0%)	156(71.23%)	57 (26.02%)	6 (2.73%)	<0.01
No married	152 (41.0%)	79 (51.97%)	69 (45.39%)	4 (2.63%)	
**Age**					
16–25	74 (19.9%)	46 (62.16%)	27 (36.48%)	1 (1.35%)	<0.001
26–35	110 (29.6%)	65 (59.09%)	40 (36.36%)	5 (4.54%)	
36–50	168 (45.3%)	114 (67.85%)	44 (26.19%)	10 (5.95%)	
50–60	16 (4.3%)	4 (25%)	11(68.75%)	1 (6.25%)	
More than 60	3 (0.8%)	1(33.3%)	1(33.3%)	1(3.33%)	
**Education**					
Less than secondary	11 (3.0%)	9(97.81%)	1(9.09%)	1(9.09%)	0.035
Diploma / Secondary	34 (9.2%)	19 (55.88%)	10(29.41%)	5(14.70%)	
Bachelor	148 (39.9%)	102(68.91%)	45(30.40%)	1(0.67%)	
Postgraduate	178 (48.0%)	127(71.34%)	47(26.40%)	4(2.24%)	
**Living Areas**					
Urban	297 (80.1%)	192(64.64%)	99(33.33%)	6(2.02%)	<0.001
Rural	74 (19.9%)	58(87.37%)	14(18.91%)	2(2.70%)	
**Health status**					
Excellent	292 (78.7%)	201(68.83%)	85(29.10%)	6(2.05%)	0.025
Good	50 (13.5%)	36(72%)	12(24%)	2(4%)	
Sick	29 (7.8%)	17(58.62%)	11(37.93%)	1(3.44%)	

We conducted univariate analyses to compare the variation among participants’ characteristics based on their exposure to social media. Social media exposure was higher among women (77.77%) than men (73.16%), and higher among married (71.23%) than unmarried (51.97%) participants. Moreover, middle-aged (aged 36–50 years) had higher social media exposure than older (aged 50–60 years) and younger (aged 26–35 years) individuals. Furthermore, participants with lower levels of education (middle and high school) had less exposure to social media than did those with higher education (undergraduate and graduate). Finally, participants from rural areas (87.37%) reported a higher rate of social media exposure than did those from urban areas (64.64%). Healthy participants (72%) were significantly more likely than others to have exposure to social media.

Table 2 presents the prevalence of anxiety, depression, and social isolation, which was 47.82%, 47.57%, and 46.42%, respectively. Furthermore, multivariate analyses indicated that the possibility of anxiety, depression and social isolation was lower among males. Further, there were no age-related differences in the incidence of anxiety and social isolation among males. However, the depression rate was higher among those aged 16–25 years as compared to those aged over 60 years. Moreover, depression, anxiety, and social isolation were higher among those who did not have high school education. Participants in rural areas had a higher rate of anxiety, depression, and social isolation than did those in urban areas. However, those with lower levels of health had higher rates of anxiety, depression, and social isolation than did those with excellent health. Furthermore, it was found that participants with frequent exposure to social media had a higher level of anxiety, depression, and social isolation as compared to those with less frequent social media exposure. In this study, social media exposure was correlated with anxiety, depression, and social isolation.

**Table 2:** Prevalence of anxiety, depression and Social isolation and relevant factors.

Variables	N	Anxiety N (%)	P-value	Depression N (%)	P-value	Social isolation N (%)	P-value
**overall**	371 (100)	141 (38.14%)		148 (39.93%)		142 (39.06%)	
**Gender**							
Male	272 (73.3%)	121 (44.5%)	<0.001	123 (45.25)	<0.001	82 (30.2%)	<0.001
Female	99 (26.7%)	50) 51.25%)		51 (52.25%)		32) 33.2%)	
**Marriage**							
Married	219 (59.0%)	79 (36%)	<0.001	80 (36.8%)	<0.001	82 (37.7%)	<0.001
No married	152 (41.0%)	59 (38.8%)		46 (30%)		61 (40.2%)	
**Age**							
16–25	74 (19.9%)	28 (38%)	<0.001	30 (40.5%)	0.016	29 (40%)	0.025
26–35	110 (29.6%)	44 (38.4%)		44 (39.8%)		45 (40.75)	
36–50	168 (45.3%)	58 (34.8)		59 (35.4%)		62 (36.85)	
50–60	16 (4.3%)	6 (39.8%)		6 (38.4%)		7 (44%)	
More than 60	3 (0.8%)	1(33%)		1(33%)		1(33.5)	
**Education**							
Less than secondary	11 (3.0%)	5(45.6%)	0.035	4 (38.2%)	<0.001	5 (46.25%)	<0.001
Diploma / Secondary	34 (9.2%)	11 (32.2%)		13 (37.8%)		14 (40.15%)	
Bachelor	148 (39.9%)	52 (34.7%)		54 (36.2%)		57 (38.6%)	
Postgraduate	178 (48.0%)	69 (38.7%)		68 (36.2%)		68 (38.3%)	
**Living Areas**							
Urban	297 (80.1%)	108 (36.4%)	<0.001	112 (37.8%)	0.035	116 (39%)	<0.001
Rural	74 (19.9%)	29 (39.8%)		30 (40.6%)		28 (38%)	
**Health status**							
Excellent	292 (78.7%)	105 (35.9%)	<0.001	107 (36.5%)	0.045	110 (37.7%)	<0.001
Good	50 (13.5%)	20 (40.2%)		21 (41.2%)		21 (42%)	
Sick	29 (7.8%)	12 (42%)		12 (42%)		13 (44.25%)	
**social media exposure**							
Frequently	303 (81.67)	112 (37.2%)	<0.001	112 (39.2%)	<0.001	114 (37.6%)	<0.001
Sometimes	56 (15.09)	21 (37.0%)		22 (37%)		23 (41.8%)	
Less	12 (3.23)	3 (26.8%)		4 (29.2%)		5 (41%)	

To find the relationship between the social media exposure and anxiety, depression and social isolation, we used regression analysis and Table 3 illustrates this correlation.

**Table 3:** Regression results for outcome variables.

Dependent Variable	R	R Square	F	Sig.	Coefficients	Unstandardized Coefficients		Standardized Coefficients	T	Sig.
						B	Std. Error	Beta		
Anxiety	.368	.135	57.828	.00	(Constant)	1.365	.071		19.352	.00
					Social media exposure	.304	.040	.368	7.604	.00
Depression	.355	.126	53.217	.00	(Constant)	1.453	.066		21.974	.00
					Social media exposure	.274	.038	.355	7.295	.00
Isolation	.342	.117	48.931	.00	(Constant)	1.286	.042		30.426	.00
					Social media exposure	.168	.024	.342	6.995	.00

Dependent Variable: Anxiety, Depression, Isolation.

Predictors: (Constant), Social media exposure.

Table 3 reveals the value of the correlation coefficient between social media exposure and anxiety, depression, and social isolation, which were (0.368), (0.355), and (0.342), respectively, and all correlation coefficients were significant at P-value (0.01). The table also shows that social media exposure has a relationship with the dependent variables (anxiety, depression, and social isolation) with a percentage variance of (0.135), (0.126), (0.117).

The results of simple linear regression analysis showed that the regression model was significant between the independent variable and the dependent variables. The level of significance for the P-value was less than (0.05). social media exposure has a variance ratio of (0.135), (0.126), (0.117), and this can also be deducted from the value of t and its significance on anxiety, depression, and social isolation.

## Discussion

The last national survey in Saudi Arabia indicated a prevalence of 23%, 6%, and 5.6% for anxiety, depression, and social isolation, respectively [58]. In comparison, our cross-sectional study on a Saudi sample during the COVID-19 outbreak found higher prevalence rates for these variables (38.14%, 39.93%, and 39.6%, respectively). These results are consistent with those of previous studies, indicating that not only general health issues but also pandemics or epidemics (including COVID-19 [18, 59–63], Ebola [64–66], and SARS outbreaks [67–69], can aggravate mental health issues in individuals.

Social media has been one of the primary sources of up-to-date COVID-19 information [15, 70]; a substantial proportion of our respondents (64%) were frequently exposed to social media. This current finding similarly assure previous finding which Saudi population is ranked within top 20 population using internet and social media compared to other population [42].This is often associated with a high risk of anxiety, depression, and social isolation [18, 34, 70, 71].

Findings of single studies related to the effects of social media on mental health in reality were contradictory [27]. It is possible that the use of social media in general may not have an impact on the mental health [72, 73]. But, during the outbreak of the pandemic, misleading information and false reports spread via social media. It raised concerns and anxiety among many users [13, 74] and may have confused and distracted people, while causing harm to their mental health [75]. Moreover, the spreading of misleading information is the most infectious disease resulting to more health problems including anxiety, depression and loneliness [12]. Besides, several individuals expressed negative feelings (such as anxiety, stress, fear, and nervousness) via social media, which may have increased similar feelings in others [76]. Hence, the WHO’s Informatics team have been collaborating with communications divisions worldwide to provide accurate information to a broader audience [77]. Furthermore, the steps taken by the Saudi Arabia—such as the closure of schools, universities, Umrah, Hajj, domestic and international aviation, and other precautionary measures—were beneficial in limiting the spread of the virus and such measures gathered praise from the WHO. However, increasingly stringent measures in the Kingdom may induce more serious mental health problems among the Saudi population.

## Conclusions

Our findings showed a significant prevalence of mental health problems within the Saudi society due to the COVID-19 pandemic; moreover, these issues are positively associated with the significant and frequent exposure to social media during the outbreak. Our results indicated that the Saudi Arabian government should pay more attention to the general population’s mental health during the COVID-19 pandemic. The Saudi government was the first to implement precautionary measures to protect its citizens, which contributed significantly to the low levels of active cases or mortality as compared to several other developed countries. Moreover, the Saudi government has been providing mental health services through various channels, including electronic applications, hotlines, online counseling and courses, and outpatient consultation. [78] However, depression, anxiety, and social isolation need to be addressed more concertedly in order to prevent their effects on infected individuals’ wellbeing. The Saudi government has also imposed numerous financial penalties and imprisoned those who published inaccurate and misleading information on social media. We suggest that, in addition to upscaling the legal actions related to the publishing of misleading COVID-19 information, the Kingdom needs to monitor social media, filter out false information, and promote the spread of accurate information through cooperation with the WHO.

### Limitations of the study

Our study has a few limitations. First, it is a cross-sectional study, which presents challenges in terms of clarifying the causal relationships between social media exposure and mental health. This can be addressed in future longitudinal studies, including case studies and cross-control studies. Furthermore, this is an intermediate sample; the survey was conducted online, which enabled rapid assessment. However, the bias created by the limited representation of elderly citizens could have affected our results. Finally, the study’s findings can only be generalized to similar sample populations and societies.

## Supporting information

S1 File (ENL)
